# Measuring Teacher Job Satisfaction: Assessing Invariance in the Teacher Job Satisfaction Scale (TJSS) Across Six Countries

**DOI:** 10.5964/ejop.v13i3.1389

**Published:** 2017-08-31

**Authors:** Alessandro Pepe, Loredana Addimando, Guido Veronese

**Affiliations:** a Department of Human Sciences "R. Massa", University of Milano-Bicocca, Milan, Italy; b Department of Teaching and Learning, University of Applied Sciences and Arts of Southern Switzerland, Locarno, Switzerland; Webster University Geneva, Geneva, Switzerland

**Keywords:** teacher job satisfaction, job satisfaction scale, measurement invariance across countries

## Abstract

Work and organizational psychology has long been concerned with measuring job satisfaction in organizational contexts, and this has carried across to the field of education, leading to a research focus on the work-related satisfaction of teachers. Today, a myriad of organizations continue to assess employees’ job satisfaction on a routine basis (Liu, Borg, & Spector, 2004). Unfortunately, a sort of balkanization of the field has resulted in the production of dozens of specific measurement tools, making it difficult to cross-compare samples and contexts. The present paper tested the measurement invariance of the Teacher Job Satisfaction Scale (TJSS) in six international cohorts (Netherlands, United States, Russia China, Italy and Palestine) of in-service teachers (N = 2,819). Confirmatory factor analysis and multi-group invariance tests were applied. The TJSS-9 displayed robust psychometric proprieties and no substantial departures from measurement invariance (configural and metric). Future research is required to further test equivalence across additional countries, with view to developing a truly international tool for measuring job satisfaction in teaching.

The term job satisfaction usually refers to the extent to which employees like the components of their job (Spector, 1997). Over time and across different paradigms, other definitions of the construct have also been adopted, sometimes in parallel with one another. A brief and non-exhaustive summary of these includes: Locke’s (1969) idea of a pleasurable or positive emotional state resulting from the appraisal of one's job or job experiences; Vroom’s (1964, 1982) focus on workers’ emotional orientation towards their job; Milkovich and Boudreau’s (1997) definition of job satisfaction as a pleasurable response to job contents; versus Schultz’s (1982) proposal that job satisfaction is simply employees’ psychological disposition towards their work. Despite the variations in this broad range of available definitions, the majority of them share the idea that job satisfaction is essentially an affective and positive job-related reaction to the workplace (Worrell, Skaggs, & Brown, 2006) that translates into how people feel about their work (Irving & Montes, 2009).

Independently of the ontological debate on how the construct should be conceptualized, countless organizations have gone on routinely assessing employees’ job satisfaction with a view to optimizing the management, training and retention of human resources (Liu, Borg, & Spector, 2004).The reasons for which job satisfaction continue to empirically intrigue managers, practitioners and researchers are easily listed, and are mainly linked to the awareness that “happy employees” can lead organizations to prosper more (and not only in terms of economic returns). In practice, job satisfaction is positively associated with organizational citizenship behaviors (Foote & Tang, 2008; Swaminathan & Jawahar, 2013), enhanced work environments (Newsham, Jay Brand, Veitch, Aries, & Charles, 2009), improved worker health (Faragher, Cass, & Cooper, 2005) and more efficient performance (Mafini & Pooe, 2013). In addition, job satisfaction is positively associated with work-related characteristics such as administration control, teaching competence and organizational culture (Ma & MacMillan, 1999). Conversely, employees’ job satisfaction is inversely associated with general (Hanebuth, 2008) and injury-related absenteeism (Drakopoulos & Grimani, 2013), intention to leave the workplace (MacIntosh & Doherty, 2010; Tschopp, Grote, & Gerber, 2014), counterproductive interpersonal and organizational behaviors (Mount, Ilies, & Johnson, 2006), job-related stress (Boudreaux, Mandry, & Brantley, 1997), psychological distress (Moen, Kelly, & Lam, 2013) and biological markers of ill-health (such as higher levels of inflammatory cytokines and other lymphocytes; Amati et al., 2010). Such negative effects on the teaching professions are crucial since, for instance, job related stress is negatively related with students’ academic achievement (Banerjee & Lamb, 2016; Kalyva, 2013).

Similarly to the construct of job satisfaction itself, the correlates and determinants of employee satisfaction have been framed differently within different theoretical paradigms. Studies about satisfaction broadly divide into two main research traditions. One line of enquiry has seen job satisfaction as largely influenced by internal and personal factors [such as an individual’s cultural background (Kwantes, 2010), level of education (Ganzach, 2003), expectations (Vroom, 1964) or perceptions of equity (Carrell & Dittrich, 1978). Maslow’s (1970) hierarchical theory of needs and Herzberg, Mausner, and Snyderman’s (1959) two-factor theory of job satisfaction are representative of this first tradition. Alternative lines of enquiry have viewed job environment and conditions and the specific contents of the job [such as pay (Diener & Seligman, 2004), organizational atmosphere (Okpara & Wynn, 2008), promotion of inequality by management (Card, Mas, Moretti, & Saez, 2012) as playing a stronger role than personal attributes in determining levels of job satisfaction. Hackman and Oldham’s (1976) job characteristics model and Karasek’s (1979) job demand-control model both belong to this second school of thought.

This lack of theoretical agreement regarding the underlying theoretical constructs has been mirrored in the production of dozens of dimension- and job-specific measurement tools, generating a sort of *balkanization* of this research field. Furthermore, the existing cross-cultural research on the topic has not contributed to unifying the debate on measures of job satisfaction. Indeed, although the first step in all organizational interventions aimed at improving employee satisfaction is to establish the existing level (Wright, Gardner, Moynihan, & Allen, 2005), one of the major difficulties for human resource professionals and researchers is still establishing how to measure employee job satisfaction in a reliable and relatively unbiased manner (Rynes, Colbert, & Brown 2002). A means of overcoming the current impasse may be the development of multi-lingual studies with a greater focus on ecological validity (Van de Vijver & Hambleton, 1996) and on assessing the degree to which the measured constructs overlap across the populations of interest (Hambleton, 1994).

With this goal in mind, the present study analyzed the efficacy of the Teacher Job Satisfaction Scale (TJSS) in assessing job satisfaction across a large sample of in-service primary teachers (*N* = 2,819) from six different countries in order to develop a reliable measurement model suitable for quick and large-scale administration in both applied and research contexts across different cultural and linguistic settings (American English, Russian, Arabic, Italian and Cantonese). By testing the structure of different linguistic versions of the Teacher Job Satisfaction Scale, we expected to obtain a reasonably stable and invariant measurement model representing a shared underlying structure of job satisfaction.

## The Teacher Job Satisfaction Scale: Developing the Questionnaire

### On the Dimensionality of Satisfaction Measures: Does Size Matter?

In 1997, Wanous, Reichers, and Hudy conducted a study on job satisfaction, concluding that job satisfaction is best assessed using a single-item measure. In another classic study, Scarpello and Campbell (1983) similarly suggested that the best way to evaluate job satisfaction is to ask workers to rate one item on a 5-point scale, namely “How satisfied are you with your job?”. These authors advocated the adoption of single-item measures of job satisfaction for three main reasons: (a) single measures of satisfaction were highly correlated with composite measures (i.e., scales); (b) single items take up less space, are more cost-effective and may feasibly be used to monitor satisfaction on a routine basis; (c) single-item measures are more popular with those administering them and more likely to be completed by employees. In addition, it has been debated whether respondents may not provide lower ratings of job satisfaction as an artifact of long and multi-faceted measures. It is possible that a long set of items may lead respondents to evaluate aspects of their job that would otherwise be marginal to their overall evaluation. A final advantage offered by the adoption of general single-item measures of job satisfaction is that they overcome the issue of job-specific measures given that they measure generic job satisfaction (Nakata, Irie, & Takahashi, 2013).

Although these arguments in favor of a single-item measure are quite compelling, especially in relation to dynamic and complex environments such as schools where time is often a crucial resource, researchers have appeared to ignore them, rarely adopting generic one-item instruments to measure job satisfaction. A review of widely-used measures of job satisfaction (van Saane, Sluiter, Verbeek, & Frings-Dresen, 2003) revealed that the majority of ready-to-administer questionnaires are multidimensional, and that even those that assess a single general dimension of satisfaction do so via multi-item scales. For instance, the Job Descriptive Index (JDI, a very popular questionnaire in organizational science; Leong & Vaux, 1992) is composed of 18 items. The Job Satisfaction Survey (Spector, 1985) measures nine facets of satisfaction via 36 items, while the Minnesota Satisfaction Questionnaire (Hirschfeld, 2000) assesses 20 aspects of job satisfaction via 100 items. Similarly, the Teacher Job satisfaction questionnaire (Lester, 1987) assessed nine different domains of job satisfaction: supervision, colleagues, working conditions, pay, responsibility, work (itself), advancement, security, and recognition. In all these cases, a multi-dimensional approach is preferred given that multiple-item measures: (a) may be easily evaluated using standard psychometric indicators (e.g., internal reliability); (b) may be used in structural equation modelling approaches; (c) offer a more structured approach to unpacking the construct of job satisfaction (Nagy, 2002). In addition, those who support the use of multi-item measures do not view potentially lower job satisfaction ratings due to the inclusion of multiple dimensions as a ‘“true” bias, arguing in contrast that such scores represent a better grounded evaluation of workplaces and, consequently, are more reliable subjective measures. In our view, both approaches have something to offer to the theory and practice of measuring job satisfaction in workplaces. On the one hand, there is an evident need for compact and easy-to-administer research tools that may easily be applied in real-life settings. In our experience, when a public or private organization agrees to participate in a study of employee job satisfaction, both respondents and management tend to perceive long batteries of item as a meaningless burden. On the other hand, there is an equally self-evident need for valid and reliable measures in the social science field. Today, the leading scientific journals implicitly require published research to have been conducted using multiple-item scales (Drolet & Morrison, 2001). In devising the Teacher Job Satisfaction Scale, we took both these aspects into account, leading us to develop our questionnaire in the spirit of an old and well-known Latin adage: *in medio stat virtus^i^*. Thus, in constructing the Teacher Job Satisfaction Scale, we attempted to trade off the efficiency of adopting single-item methods against the efficacy of adopting multi-item measures, in such a way that the drawbacks of one method were offset by the advantages of the other and vice versa. Specifically, in the interests of achieving this ideal balance, we: (a) included a single overall item for each facet of job satisfaction in our measurement model; (b) balanced each sub-scale of the TJSS by including at least two specific items for each facet; (c) ensured that the overall questionnaire–though brief–was long enough to enable evaluation of its psychometric proprieties.

### On the Operationalization of Job Satisfaction: Key Domains in the Educational Context

The rationale for viewing job satisfaction in teaching as a key focus for educational research mainly concerns the benefits, for both teachers and students, that “satisfied” teachers are known to contribute to organizational performance (Heller, Rex, & Cline, 1992). It has frequently been reported that satisfied teachers display high levels of job commitment and are less at risk of leaving the profession (Gersten, 2001; Singh & Billingsley, 1996). Similarly, Klassen and Chiu (2010) confirmed the negative relationship between job satisfaction and occupational stress by demonstrating that, among primary teachers, high levels of occupational stress correspond to low levels of job satisfaction. Another study conducted in the Italian context (Caprara, Barbaranelli, Steca, & Malone, 2006) reported that job satisfaction was predicted by teachers’ self-efficacy beliefs and, in turn, it affects students’ academic achievement.

In general, it may be concluded that job satisfaction in teaching is derived from the gratification of higher order needs such as positive social relationships, rather than lower order needs (e.g., pay incentives) (Sylvia & Hutchinson, 1985). In fact, recent research shows that interpersonal relationships play a key role in the work of teachers (Van Droogenbroeck, Spruyt, & Vanroelen, 2014) and that satisfaction with positive relationships with co-workers, parents, and students mitigates some of the adverse effects of teaching work (Cano-García, Padilla-Muñoz, & Carrasco-Ortiz, 2005; Gavish & Friedman, 2010; Skaalvik & Skaalvik, 2011). These findings, along with the framework offered by Herzberg’s (1966) dual-factor satisfaction theory, which has been widely applied in research examining K-12 (i.e., the label k-12 refers to comprises the sum of primary and secondary education in the Anglophone countries) teacher satisfaction (e.g., Perie & Baker, 1997), provided robust justification for our choice of the particular facets of job satisfaction to include in the measurement model of the Teachers Job Satisfaction Scale.

Teachers’ relationship with their students was the obvious choice for the first dimension in our operational model for evaluating the job satisfaction of primary teachers. Today, there is broad consensus among researchers (e.g., Addimando 2013; Chang, 2009; Pepe & Addimando, 2013; Spilt, Koomen, & Thijs, 2011) that the most common source of work-related stress in teachers is their interaction with pupils. Negative relationships in the classroom are frequently related to difficulty with classroom management (Wubbels, Brekelmans, den Brok, & van Tartwijk, 2006), a key factor in stress and burnout later on in teachers’ careers (Tatar & Horenczyk, 2003; Veldman, van Tartwijk, Brekelmans, & Wubbels, 2013). The second dimension was also related to the social atmosphere in the work organization and, specifically, on how relations with co-workers influence employees’ job satisfaction (Ellickson & Logsdon, 2001). In particular, Luthans (2005) recommended viewing this factor as a major determinant of job satisfaction. Similarly, work by Ghenghesh (2013), suggested that the quality of teachers’ relationships with co-workers is a key variable influencing their job satisfaction. Finally, in line with current thinking about the social aspects of teachers’ work, the third dimension included in the model was satisfaction with parents. Extensive research (Fan & Chen, 2001; Houtenville & Conway, 2008; Jeynes, 2007; Jeynes, 2010) has explored the importance of parental involvement for children’s school achievement, suggesting that families should be fully included in school processes. In a recent quantitative synthesis of research about parental involvement (Castro, Exposito-Casas, Lopez-Martin, Lizasoain, Navarro-Asencio, & Gaviria, 2015), a strong general relation emerged between type of parental involvement and academic achievement, in particular with regards to develop and maintain communication with them about school activities and schoolwork and promote reading habits. On the contrary, supervision and control of homework and parental attendance of school activities do not appear to be especially related to the children’s academic achievement (p. 13). Other studies (see Szumski & Karwowski, 2012) confirmed the positive relation between engagement of parents and students‘ academic achievement and supported the thesis that in the process of placing children with disabilities, selection processes based on students’ social origin do take place (p. 1623).

## The Present Study

The aim of the present study was to test the factor structure of the Teacher Job Satisfaction Scale (TJSS), along with its measurement invariance across six subsamples of primary teachers. Given that theoretical advances on the topic of job satisfaction suggest the adoption of stable and robust quantitative tools enabling the cross-comparison of results, we tested different competing factor structures for the TJSS via standard confirmatory factor analysis (CFA) in order to estimate the scale’s psychometric proprieties in a large sample of teachers (*N* = 2,819). In particular, we evaluated four different models of the TJSS-9: unidimensional (1), three-dimensional with uncorrelated sub-scales and item-level errors not allowed to co-vary (2), three-dimensional with correlated sub-scales and item-level errors not allowed to co-vary (3) and three-dimensional with correlated sub-scales and covariance among item-level errors allowed (4). Details of the technical aspects guiding our assessment of the “best fitting baseline structure” are reported in the Method section.

Next, in order to improve the ecological validity of our findings, we explored measure invariance across all sub-samples by applying multiple-group confirmatory factor analysis (MGCFA) with a cross-validation procedure (Byrne, 2004). First, we tested the hypothesis that job satisfaction scores would be represented by three factors in all groups: satisfaction with co-workers, satisfaction with students and satisfaction with parents. To this end, configural invariance (φ^g^ = φ^g’^) (a weak type of factorial invariance test; Horn & McArdle, 1992) was measured. Next, metric invariance (∧_i,j_^g^ = ∧_i,j_^g′^) was tested by constraining all factor loadings to be equal across groups. During the third step, the scalar invariance of TJSS was specified and item intercepts (τ^g^ = τ^g’^) were set to be equal, so that latent factors means could be meaningfully compared across groups. Finally, in keeping with standard practice (for details see Cheung & Rensvold, 2002), error variance invariance (Θ_δ_^g^ = Θ_δ_^g’^) and full construct invariance (all parameters fixed to be equal) were also evaluated (see also Cavalera, Pepe, Zurloni, Diana, & Realdon, 2017; Grazzani, Ornaghi, Pepe, Brazzelli, & Reeffe, 2017; Veronese & Pepe, 2013). Measures of reliability as well as the convergent/concurrent validity of the TJSS scales with the General Health Questionnaire (GHQ-12) were also calculated. In the final section of this paper, we discuss the empirical results obtained in terms of both the implications for theoretical research of using an invariant model of satisfaction and the practical implications of adopting the TJSS-9 scale as a means of collecting information about teachers’ job satisfaction across different contexts.

## Methodology

### Participants

The sample was composed of primary teachers (*N* = 2,819) recruited in schools located in six different countries: the Netherlands (*n* = 551, 19.6%), the United States (*n* = 284, 7.8%), Russia (*n* = 169, 6.0%), China (*n* = 934, 33.2%), Italy (*n* = 724, 25.9%), and Palestine (*n* = 149, 4.1%). All teachers (100%) worked in state-run primary schools. Gender distribution was 2,394 females (85.2%) and 411 males (14.6%) (4 missing values). The characteristics of the country sub-samples are reported in Table 1 and they indicate that it was appropriate to test the structural invariance of TJSS using MGCFA cross validation procedures.

**Table 1:** Characteristics of Sub-Samples

	Gender		Tenure	
	female	male		
	*n* (%)	*n* (%)	*M*	*SD*
Netherlands	439 (79.5)	112 (20.5)	17.3	11.0
Russia	166 (98.2)	3 (1.8)	17.9	7.8
China (Hong Kong)	801 (85.6)	133 (14.4)	11.9	8.1
United States	266 (93.6)	17 (16.4)	12.8	9.6
Italy	668 (92.0)	56 (8.0)	17.7	9.6
Palestine	54 (63.8)	95 (36.2)	10.4	8.3

*Note*. According to World Bank (2014, http://data.worldbank.org/indicator/SE.PRM.TCHR.FE.ZS), general female ratios (%) in primary schools were: Netherlands (85%), Russia (99%), China (Hong Kong, 78%), United States (88%), Italy (96%), Palestine (not available).

Participation in the study was on voluntary basis, with all participants recruited on-site and surveyed at their workplaces. Questionnaires were anonymous and the data was handled collectively. Only teachers who had been in charge of their own classrooms for at least one full year at the time of the study were eligible to participate. The study was conducted following APA ethical principles and code of conduct (American Psychological Association, 2010).

### Measures

#### Teacher Job Satisfaction Scale (TJJS-9)

The Teacher Job Satisfaction Scale (Pepe, 2011) is a questionnaire aimed at measuring job satisfaction that has been specifically developed for use in educational contexts. The TJSS-9 is composed of three dimensions: satisfaction with co-workers (3 items), satisfaction with parents (3 items) and satisfaction with students’ behaviors (3 items). Items are rated on a 5-point scale (1 = *I am highly dissatisfied with this aspect of the school*, 5 = *I am highly satisfied with this aspect of the school*). For instance, the dimension “co-workers” included items such as “The quality of your relations with co-workers” or “The extent to which your co-workers encourage you and support you in your work”. Given that from the outset we were interested in developing an instrument with structural invariance, great care was taken when translating the items into the different languages, taking the “ask the same question” rule (Harkness, Van de Vijver, & Mohler, 2003) as a guideline. The first draft of the Teacher Job Satisfaction Scale was developed in English in order to facilitate its subsequent translation into other languages. For each country, two bilingual (English and target language) researchers worked separately to obtain a “local” version of the questionnaire. A third research coordinator supervised their products. All translated versions of the questionnaire were then explored by small groups of teachers (up to eight) to evaluate the target-language translation and adaptation (in terms of familiarity and specificity of meanings). Finally, all language versions (English, Dutch, Russian, Italian, Cantonese, Arab) were administered during preliminary pilot sessions. The current version of the instrument (with 9 items) was developed from an original set of 35 items loading on six different dimensions: satisfaction with all the colleagues, satisfaction with co-workers, satisfaction with management, satisfaction with parents, satisfaction with students’ behavior and responsibility. From that initial version of the questionnaire, a series of exploratory and confirmatory analyses were conducted on data from local samples, with a view to making the TJSS measurement model more robust, reliable and compact. Cronbach’s alpha values and the means of inter-item correlations for each sub-sample are reported separately in the Results section.

#### General Health Questionnaire (GHQ-12)

The General Health Questionnaire (GHQ) (Goldberg, 1972) is a family of reliable quantitative tools aimed at “detecting psychiatric disorders among respondents in both community and non-clinical settings” (Goldberg & Williams, 1988, p. 1). The GHQ-12 is one of the shorter versions of the original instrument (GHQ-30), which has often been used in the context of large-scale social surveys (e.g., by the World Health Organization) given its proven reliability and sensitivity in measuring psychological distress across multiple groups of participants. In order to assess psychological distress among teachers in the present study, we adopted the tripartite measurement model composed of anxiety (4 items), social dysfunction (6 items) and loss of confidence (2 items). Each subscale has scores ranging from 0to 3. We decided to include the GHQ-12 as a means of cross-validating the TJSS-9 scores for three main reasons: (a) there is an unusually strong positive relationship between job satisfaction and psychological health (Faragher, Cass, & Cooper, 2005); (b) specific language versions of the measure had already been translated, culturally adapted and reported in the literature for each of the countries in our study; (c) the GHQ-12 measure has been advantageously used in previous teacher studies (Pepe & Addimando, 2014) yielding satisfactory results in terms of reliability of scores and normality of distribution. Cronbach’s alpha values and the means of inter-item correlations for each sub-sample are reported separately in the Results section.

### Statistical Procedures

#### Confirmatory Factor Analysis (CFA)

The software Amos 21.0 was used to specify and assess different measurement models based on the variance-covariance matrix of the TJSS-9 scores. Prior to analysis, all variables were checked for assumptions related to factor analysis (i.e., homoscedasticity, multivariate normality, etc.). No excess skewness or other major violations to normality were found. Multivariate outliers were identified and skipped via the application of a *p* > .001 criterion for Mahalanobis’ distance. Next, the CFA method was applied (estimation method: maximum likelihood) to evaluate four different models for the overall sample: the purpose of this first set of analyses was to specify a robust and “shared” model of TJSS-9 to serve as baseline for further analysis. The first was a unidimensional solution (M1), in which all items measuring job satisfaction loaded together onto a single unidimensional factor. Then, a three-dimensional model (satisfaction with co-workers, students and parents) with uncorrelated subscales and uncorrelated item-level errors (M2) was assessed. Next, a third model (M3) allowing scales to covary with uncorrelated item-level errors (M3) was specified. Finally, a less constrained measurement model (M4) with correlated dimensions and allowing item-level errors to correlate (as suggested by Crawford and Henry, 2004) was tested. The degree to which each model fitted the empirical data was evaluated for both practical significance and statistical significance using the following goodness-of-fit indices: Root Mean Square Error of Approximation (RMSEA, the cut-off value for the RMSEA is .05 [Steiger, 2007] while more robust models require the RMSEA 90^th^ confidence interval to fall entirely below.08 [Mac Callum, Browne, & Sugawara,1996]); Standardized Root Mean Square Residual (SRMR, a value of less than .08 for SRMR is generally considered a good result [Hu & Bentler, 1999]); Normed Fit Index (NFI, the index must have a value of over .95 [Kline, 2005]).Comparative Fit Index (CFI, a cut-off point of. 95 is generally accepted [Hu & Bentler, 1999]); Tucker–Lewis Index (TLI, also known as the Non-Normed Fit Index, for a model to be accepted, the index must have a value of over .95 [Tabachnick & Fidell, 2007]).

#### Structural Invariance and Multiple-Group Confirmatory Factor Analysis (MGCFA)

When researchers aim to assess the measurement equivalence of an original questionnaire and its translated versions, MGCFA should be applied (Koh & Zumbo, 2008). The hypothesis of invariance across measures is supported when the network of relations between latent constructs and observed variables are identical across groups of interest. In other words, this type of analysis tests whether or not measurements yield measures of the same attributes under different conditions of the empirical world (Horn & McArdle, 1992). Given that we were interested in developing a ready-to-administer version of TJSS in different languages, we applied standard procedures for testing measurement invariance (Vandenberg & Lance, 2000) to groups of teachers from different countries. Such comparisons help to identify the model with the best generalization potential (Hair, Black, Babin, & Anderson 2010). In order to assess invariance, the statistical significance of the variation in χ^2^ is calculated at each hierarchical step. The hypothesis of equivalence across groups is rejected if the indexed variations are statistically significant. The cut-off points of other fit indexes (ΔCFI, ΔRMSEA, ΔSRMR) for rejecting measurement invariance were set at Δ < .01, corresponding to a *p*-level of .01 (Chen, 2007).

#### Concurrent Validity and Reliability

Finally, in order to “enhance confidence that the concept under study is being captured” (Short, Ketchen, Shook, & Ireland, 2010, p. 53), the present study used measures of psychological distress to evaluate the concurrent validity of TJSS-9 scores. Specifically, GHQ-12 scores were used as a proxy (i.e., as a negatively correlated measure of the original construct) and analysed for correlation with job satisfaction scores. A statistically significant correlation between TJSS-9 and GHQ-12 variables would imply concurrent validity. Cronbach’s *α* values and confidence intervals (CI; based on Feldt’s [1965] formula) of GHQ-12 scores were: *α* = .794, 95% CI [.781, .807].

## Results

Our first goal was to obtain a measurement model serving as baseline for the analysis of structural invariance across groups. The main descriptive values (means, standard deviations and measures of distribution) are reported in Table 2.

**Table 2:** Descriptive Statistics for TJSS-9 Items

	*M*	*SD*	Skewness	Kurtosis
The quality of your relations with co-workers	3.99	0.79	-0.64	0.60
The extent to which your co-workers encourage you and support you in your work	3.80	0.87	-0.53	0.18
Your overall satisfaction with your co-workers	3.92	0.79	-0.60	0.60
The extent to which students act in a self-disciplined manner	3.16	0.85	-0.24	-0.23
Your satisfaction with the behavior of students in your school	3.35	0.81	-0.48	0.05
Your overall level of satisfaction with student discipline in your school	3.29	0.80	-0.42	-0.06
The degree of interest shown by parents in the education of their children	3.31	0.91	-0.41	-0.21
The extent to which parents are supportive of the school and its programs	3.22	0.89	-0.32	-0.13
Your overall level of satisfaction with parents where you work	3.32	0.88	-0.36	-0.10

The results of confirmatory factor analysis for the nine items of the Teacher Job Satisfaction Scale are reported in Table 3.

**Table 3:** Goodness of Fit Indices for CFA Models of the TJSS-9 (N = 2,819)

	χ^2^	*df*	RMSEA	SRMR	NFI	NNFI	CFI
Unidimensional (M1)	8,195.2	27	.27	.15	.61	.48	.61
Three independent dimensions(M2)	2,287.4	27	.14	.26	.89	.85	.89
Three dependent dimensions (M3)	151.2	24	.04	.02	.99	.99	.99
Three dependent dimensions with correlated item-level errors (M4)	128.5	22	.03	.02	.99	.99	.99

**Figure 1:**
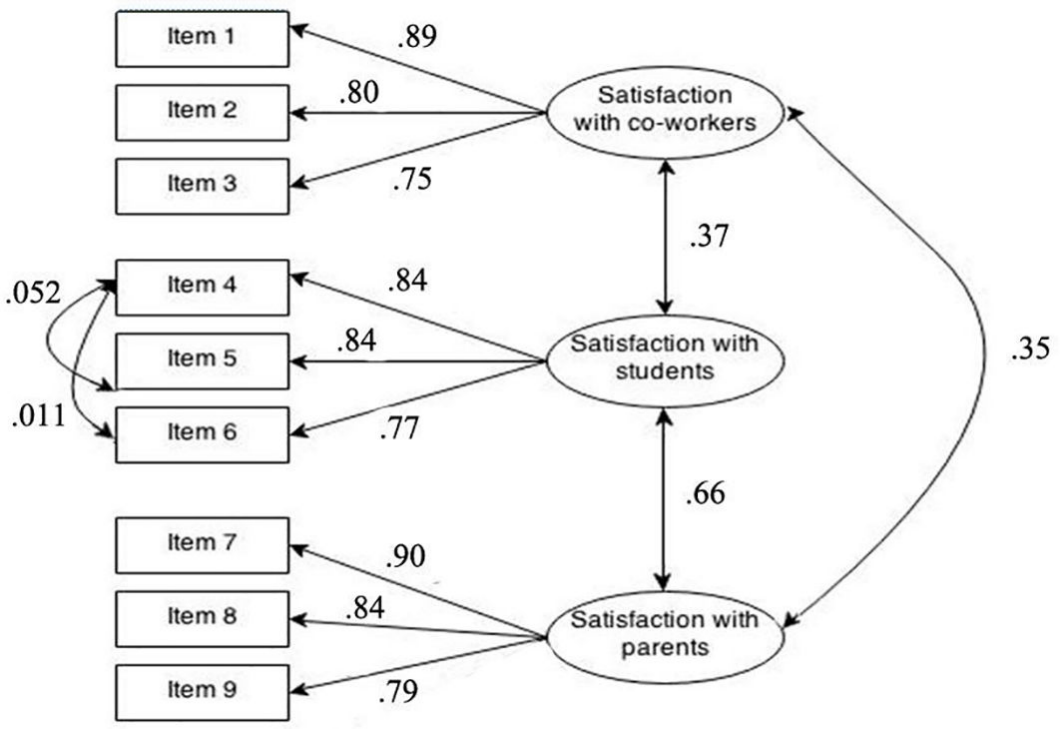
TJSS Model Measurement (M4).

The one-dimensional model (M1) yielded a very poor fit with the data, as reflected in all the fit indexes. This model displayed null statistical and practical significance and so the hypothesis of mono-dimensional measurement of job satisfaction was rejected. The second model representing three independent dimensions of satisfaction was also rejected: once again, the fit indexes were very low, χ^2^(27) = 2,287.4, *p* < .001: RMSEA = .143; SRMR = .26; CFI = .89; NNFI = .85; NFI = .89. Model M 3 representing satisfaction as comprising three co-dependent dimensions yielded satisfactory fit indexes, suggesting that it should be accepted. The model displayed both statistical and practical significance, CFI = .993, NNFI = .991; NFI = .994). Finally, the last model (M4) obtained equally robust fit indexes, leading us to adopt it as our baseline for assessing multi-group structural invariance. The relationships among items and dimensions of satisfaction are reported in Figure 1.

The baseline model confirmed the original tripartite structure of the questionnaire: satisfaction with co-workers, satisfaction with students and satisfaction with parents. The first dimension is composed of the following items: (a) the quality of your relations with co-workers (λ_11_ = .89, *SE* = .011); (b) the extent to which your co-workers encourage you and support you in your work (λ_21_ = .80, *SE* = .019); (c) your overall satisfaction with your co-workers (λ_31_ = .75, *SE* = .024). The second dimension is made up of a further three items: (d) the extent to which students act in a self-disciplined manner (λ_42_ = .84, *SE* = .015); (e) your satisfaction with the behavior of students in your school (λ_52_ = .84, *SE* = .015); (f) your overall level of satisfaction with student discipline in your school (λ_62_ = .77, *SE* = .023). Finally, the third dimension comprises the three items (g) the degree of interest shown by parents in the education of their children (λ_73_ = .90, *SE* = .010); (h) the extent to which parents are supportive of the school and its programs (λ_83_ = .84, *SE* = .015); (i) your overall level of satisfaction with parents where you work (λ_93_ = .79, *SE* = .021). Factor loadings on the individual items (λ_i,j_) all displayed strong practical significance, λ values were all above the recommended cut-off point (λ > .3) at a good level of statistical significance and with low standard errors. The p values for factor loadings were < .001 in all cases. In addition, the model specified relationships among latent variables. In particular, satisfaction with parents and satisfaction with students displayed a strong, statistically significant and positive correlation (φ_32_ = .66, *p* < .001) On the contrary, the variable satisfaction with co-workers appeared to be less strongly related to the other two components (φ_12_ = .37, *p* < .001, φ_13_ = .35, *p* < .001) of job satisfaction. Finally, only two co-variance errors (θ_i,j_) were included in the model (θ_45_ = .052, θ_46_ = .011). This seemed to be appropriate given that the parameters did not alter the structure of the λ parameters previously hypothesized in M2 and M3 (Bagozzi, 1983) and they represented non-random measurement errors linked to local sample specificities (Byrne, Shavelson, & Muthén, 1989).

The subsequent step involved the adoption of MGCFA analysis to assess the measurement invariance of TJSS-9 across the country subsamples of teachers (Table 4). The multi-group test for configural invariance provided strong numerical support for accepting the hypothesis of configural equivalence (ΔX^2^ and all other differences between fit indexes were acceptable) among factor structures, meaning that participants from different groups conceptualized the construct of job satisfaction in the same way. This was the weakest test of factorial invariance and the outcome suggested that the same pattern of free and fixed parameters applied to each group of teachers.

**Table 4:** Results of Tests of Invariance in TJSS-9, Δ Values of Indexes Are Reported

Model	X^2^	*df*	ΔX^2^ (*p*)	RMSEA	SRMR	NFI	NNFI	CFI
Baseline model	128.5	22	-	.018	.035	.99	.99	.99
1. Configural invariance (M4a)	265.6	192	137.1 (.97)	.017	.028	.99	.99	.99
2. Metric invariance (M4b)	281.9	162	16.24 (.98)	.019	.033	.98	.98	.98
3. Scalar equivalence (M4c)	1,584.4	207	1,318.7 (.001)	.043	.064	.90	.91	.91
4. Error variance invariance (M4d)	2,008.9	237	424.4 (.001)	.045	.106	.87	.90	.89
5. Complete invariance (M4e)	2,483.66	292	474.7 (.001)	.046	.113	.84	.89	.86

*Note.* **p <.001.

We next tested item-level metric invariance (∧_g_ = ∧_g′_) by specifying equal item-factor loadings across groups. This test allowed us to assess whether item ratings could be compared across groups and whether item differences indicated disparity in the underlying constructs. Goodness-of-fit indexes provided robust support for the metric invariance of TJSS-9 across educational contexts, suggesting strong factorial invariance of TJSS-9. The difference between M4b and M4a in terms of Δχ^2^was not statistically significant. The hypothesis of invariant factor loadings patterns was therefore accepted. Next, scalar invariance (τ^g^ = τ^g’^) and error variance-invariance (Θ_δ_^g^ = Θ_δ_^g’^) were evaluated. In the first case, the results did not support scalar invariance of the TJSS-9, revealing that the teachers in the different groups did not have equal intercepts for the observed variables. In the latter case, the groups displayed unequal levels of variance in relation to the invariant items. Consequently, the test for full invariance led to similarly unsatisfactory results and to rejection of the hypothesis of full invariance.

### Reliability Analysis and Convergent Validity

The main descriptive statistics for the TJSS-9 sub-scale scores of each national group are summarized in Table 5. In general, all the dimensions of job satisfaction were reliable and displayed normal distribution. With regard to convergent validity, zero-order correlations for the overall sample confirmed that teachers’ psychological distress scores as measured by the GHQ-12 were negatively and moderately correlated with satisfaction with coworkers (*r* = -.179**), satisfaction with students (*r* = -.233**) and satisfaction with parents (*r* = -.180**). The magnitude of these correlations were in line with other studies exploring correlations between job satisfaction and GHQ-12 scores [e.g., Amati et al. (2010) found a correlation of -.235 in nurses, Kawada and Yamada (2012) found a correlation -.275 in general workers].

**Table 5:** Descriptive Statistics and Internal Consistency of TJSS-9 Subscale for Each Sub-Sample

	Satisfaction with coworker				Satisfaction with students				Satisfaction with parents			
	*M*	*SD*	alpha	skewness	*M*	*SD*	alpha	skewenss	*M*	*SD*	alpha	skewness
Netherlands	12.39	1.87	.789	-.657	10.70	1.91	.823	-.537	10.63	2.21	.822	-.660
Russia	12.41	2.07	.797	-.824	9.52	1.96	.722	-.391	9.82	2.35	.787	-.520
China (Hong Kong)	11.68	1.58	.848	-.002	9.59	1.87	.898	-.284	9.94	1.89	.901	-.279
United States	12.69	2.17	.884	-1.090	10.00	2.44	.872	-.538	9.71	3.14	.937	-.441
Italy	11.52	2.47	.856	-.703	9.91	2.14	.810	-.433	9.89	2.37	.843	-.255
Palestine	8.26	1.28	.801	-1.820	6.85	1.71	.785	-.301	6.55	1.74	.789	-.155

## Conclusion

The present study evaluated the measurement invariance of the Teacher Job Satisfaction Scale (TJSS-9) in six cohorts of primary teachers at state-run schools in six countries. To this end, confirmatory factor analysis, multi-group comparison, internal consistency and convergent validity tests were conducted. Overall, the results of the CFA supported a measurement model of job satisfaction in educational contexts covering three domains explaining 79.5% of variance in teachers’ overall work-related satisfaction: satisfaction with students, satisfaction with co-workers and satisfaction with parents. The baseline structure is composed of nine items and two-correlated item-level errors. The inclusion of covariance among item-level errors into the final baseline model did not substantially alter the parameters previously estimated (Bagozzi, 1983, p. 2) and they represented non-random error determined by the peculiar characteristics of samples (Fiorilli et al., 2015; Veronese & Pepe, 2017). The latent factors displayed excellent internal and good convergent validity. The observed cumulative indicators were normally distributed, meaning that the TJSS-9 represents an excellent screening tool for assessing primary teachers’ levels of job satisfaction. With regard to measurement invariance, the results of the MGCFA fulfilled the requirements for construct equivalence and measurement unit equivalence. Therefore, the different language versions of the TJSS-9 (American English, Russian, Arabic, Italian and Cantonese) may be said to display a shared factor model with similar underlying meanings across groups, implying that factor variance may be compared across groups. On the other hand, the present study did not support full scale equivalence, meaning that groups scores should be compared with extreme caution. In addition, the present study adopted a measure of psychological distress (General Health Questionnaire; Goldberg, 1972) to support convergent validity. From this point of view, further researches adopting the TJSS-9 will require the use of other measures for eventually evaluating discriminant validity (e.g., turnover intentions) of the scale. Given these outcomes, we propose the following guidelines for the future use and development of the TJSS-9. First, from a theoretical point of view, our study confirms (in the educational field specifically) the importance of social and interactional aspects of work in determining the job satisfaction of the workforce. Following in the tradition of studies using social information processing approaches (Salancik & Pfeffer, 1978), our findings support the idea that job attitudes and perceptions are influenced by social cues picked up directly from the work environment. The pattern of parameters, the relations among items and latent factors, as well as the amount of explained variance observed in our study all appear to point in this direction. This does not mean that job tasks or dispositional dimensions are not implicated (or should be excluded from investigations of job satisfaction in teaching), but the inclusion of heterogonous cohorts from different countries and educational contexts in the present research design provides a strong basis for viewing such aspects as hierarchically subordinated to social and relational aspects. Furthermore, the basic invariance of the TJSS-9 measurement model across groups observed in our data, provides valuable evidence for relatively stable and, perhaps, pan-cultural dimensions of teachers’ job satisfaction.

Second, from a methodological point of view, the idea that job satisfaction is best measured by a single-item measure is only partially borne out by our research and findings. From our experience in developing the TJSS-9, we conclude that overall indicators (including for specific dimensions) of job satisfaction help to evaluate and comprehend the constructs under study. However, it appears that it is also of benefit to include a small number of more specific items with a view to creating a more comprehensive framework allowing the exploration of concurrent effects. In fact, one of the possible explanations for the TJSS-9’s excellent psychometric proprieties is the inclusion of specific indicators completing and reinforcing the overall measures of job satisfaction.

Third, from a practical point of view, the administration method (paper and pencil), respondent burden (less than 10 minutes) and ease of interpretation score characterizing this instrument all encourage its adoption across different settings (e.g., for routine screening) and national contexts. Consequently, the TJSS-9 may be classed as a short and user-friendly measure of job satisfaction representing a valuable resource for professionals and practitioners whose aim is to collect data as easily as possible while avoiding overburdening individuals working in dynamic organizations (such as schools).

### Limitations

As is usually the case in research, the current work also presents limitations that warrant discussion. First, the research design was cross-sectional and a further interesting development will therefore be to longitudinally track the job satisfaction patterns of different cohorts of teachers. Second, scalar and full invariance were not confirmed for the measure under study, meaning that great caution should be applied to the comparison of TJSS-9 scores across cultural groups. Third, we administered the questionnaire to convenience samples of in-service primary teachers working in state-run schools only. Although we tried to compensate for sampling errors by increasing the size of the samples, the scope for generalizing our findings to other teacher populations remains limited. Then, another limitation is about the content of the TJSS-9. Specifically, the items included into the model of measurement are aimed at assessing specifically satisfaction with social relationships (students, parents and colleagues). Other important aspects that contribute to job satisfaction (e.g., organizational culture, climate, pay, perception of autonomy, etc.) were not included in the questionnaire. Consequently, in order to get a complete evaluation of job satisfaction it is important to place side by side TJSS-9 with other complementary measure of job satisfaction. Finally, we only had the opportunity to translate and administer the questionnaire in six different language versions: there is still a long way to go before the goal of a truly international tool for evaluating teachers’ job satisfaction may be achieved.
